# Physiological Functions of Freshwater Clam Extracts and the Exploration of Their Bioactive Compounds

**DOI:** 10.3390/foods15111870

**Published:** 2026-05-25

**Authors:** Kyoko Kuwano, Masahiro Hata, Miki Umeki, Satoshi Mochizuki, Hiroaki Oda, Takao Shimazoe

**Affiliations:** 1 Sasaki Foods Co., Ltd., Ōita City 879-0615, Japan; k-kuwano@sasakishokuhin.co.jp (K.K.); m-hata@sasakishokuhin.co.jp (M.H.); 2 Faculty of Food Science and Nutrition, Beppu University, Beppu 874-8577, Japan; umemiki@nm.beppu-u.ac.jp; 3 Faculty of Education, Oita University, Ōita City 870-1192, Japan; smochi@oita-u.ac.jp; 4 School of Health and Human Life, Nagoya Bunri University, Nagoya 492-8520, Japan

**Keywords:** *Corbicula fluminea*, freshwater clam, hepatic injury, lipid metabolism, alcohol metabolism, hypercholesterolaemia, metabolic syndrome, MASLD, MASH

## Abstract

Freshwater clams (*Corbicula* spp.), commonly known as *shijimi* in East Asia, have long been valued for their functional and nutritional properties. In this review, we summarise the physiological effects of hot water extracts derived from Taiwanese freshwater clams (FCE), particularly in relation to metabolic syndrome and other lifestyle-related disorders. Traditionally, *shijimi* has been used to alleviate hepatic dysfunction and symptoms associated with alcohol consumption. FCE significantly suppresses galactosamine-induced increases in serum aspartate aminotransferase and alanine aminotransferase. In addition, FCE reduces alcohol-induced hepatic lipid accumulation and lowers hepatic cholesterol levels. FCE also influences alcohol metabolism: animals receiving FCE exhibit lower blood alcohol concentrations and a faster rate of alcohol clearance following ethanol administration. These findings suggest that *shijimi* may protect against alcohol- or drug-induced liver damage, potentially by enhancing alcohol metabolism. Beyond its role in liver protection, *shijimi* has been associated with the alleviation of jaundice, possibly through increased bile secretion linked to improved cholesterol homeostasis. Supporting this, studies using models of exogenous hypercholesterolemia show that FCE lowers both serum and hepatic cholesterol levels in a dose-dependent manner. Overall, traditional claims regarding the hepatoprotective effects of *shijimi* are increasingly supported by mechanistic and molecular evidence. This is the first clarified review of the various effects of *shijimi*.

## 1. Introduction

Metabolic syndrome is a cluster of metabolic abnormalities, including obesity, insulin resistance, dyslipidaemia, hypertension, and impaired glucose tolerance, which arise from the interaction of multiple genetic predispositions and environmental factors, such as physical activity and dietary habits. The global burden of cirrhosis attributable to metabolic dysfunction-associated steatotic liver disease (MASLD) increased from 1990 to 2021, reaching 1.267 billion cases (95% confidence interval [CI]: 1.158–1.380 billion) and 97,400 deaths (95% CI: 69,500–130,200) in 2021 [1]. The liver is referred to as a “silent organ”, as pathological changes are often difficult to detect. MASLD and metabolic dysfunction-associated steatohepatitis (MASH) are considered hepatic phenotypes of metabolic syndrome. Although MASLD has not traditionally received significant attention, substantial lifestyle changes have led to an increasing prevalence of MASLD and MASH. MASLD can lead to liver fibrosis, cirrhosis, and hepatocellular carcinoma, and treatment options for cirrhosis are limited once it has developed, making early identification and intervention crucial [1]. Consequently, preventive and therapeutic strategies have become a focus of growing interest.

Over the past several decades, numerous valuable natural products have been isolated from terrestrial organisms and developed as pharmaceuticals. However, as the metabolic pathways and chemical space of terrestrial species have been extensively characterised, the identification of compounds with genuinely novel scaffolds from these sources has become increasingly limited. In contrast, aquatic organisms exhibit remarkable biodiversity and are considered an abundant yet underexploited source of bioactive compounds, thereby becoming a focal point in marine biotechnology [2]. Owing to distinct metabolic routes and limited prior investigation, aquatic species are believed to produce many novel metabolites. Among these, deep-sea organisms remain largely inaccessible; however, advancements in manned submersibles and remotely operated vehicles have enabled systematic exploration of these environments, leading to the identification of several promising species [3]. Despite this progress, the cultivation and harvesting of deep-sea organisms remain technically challenging because of their highly specialised environmental requirements. In contrast, shallow-water species are more amenable to propagation, with established aquaculture techniques available for many species. Systematic searches for bioactive metabolites in edible shallow-water species, such as shellfish, which have been harvested for food for centuries, remain scarce.

Historically, shellfish have been used in traditional medicine and ascribed various therapeutic effects [4,5]. For instance, the Māori in New Zealand traditionally consume green-lipped mussels raw as a general tonic [5], whereas in Okinawa, the thorny oyster *Spondylus varius* has been used empirically to treat hepatic disorders [6]. The *Bencao Gangmu*, one of the earliest comprehensive Chinese materia medica, records that freshwater clams “dispel acute heat, brighten the eyes, promote urination, relieve fever, oedema, and damp toxicity, and counteract the jaundice caused by alcohol; their broth quenches thirst”. Accordingly, clam flesh and broth are traditionally used for hepatic support in China, Taiwan, Japan, and other East Asian countries. Despite this ethnopharmacological foundation, scientific studies on the bioactivity of shellfish remain limited.

Shellfish are rich in amino acids, and interspecies variations contribute to distinct physiological effects, in addition to nitrogen provision. Taurine, a sulphur-containing amino acid abundant in many shellfish, enhances lipid catabolism [7]. Glycine promotes lipid utilisation [8,9] and confers hepatoprotective effects [10]. Compared to fish extracts, shellfish extracts contain lower levels of histidine and higher concentrations of glycine, alanine, glutamic acid, arginine, proline, aspartic acid, and taurine [11]. In addition to amino acids, sulphated polysaccharides derived from abalone exhibit immunomodulatory, antitumour, and antioxidant activities [12,13]. Furthermore, oysters and short-neck clams improve lipid metabolism [14,15], an effect partly attributed to their triacylglycerols, which are rich in polyunsaturated fatty acids that suppress hepatic lipogenic enzymes [15].

The freshwater clam (*Corbicula* spp.), a bivalve cited in the *Bencao Gangmu* and long recognised in Japan for its hepatoprotective properties, contains high levels of the amino acid ornithine [16] and exhibits intrinsic hepatoprotective activity [17]. Freezing clams further increases their ornithine content [18] and concurrently reduces acorbine, a naturally occurring tripeptide, β-Ala-Orn-Orn [19]. Similarly to other marine-derived foods, *Corbicula* lipids are rich in polyunsaturated fatty acids [15] and contain plant sterols that inhibit cholesterol absorption [20]. As animals cannot synthesise plant sterols, clams likely accumulate them from phytoplankton. A polyoxygenated sterol [21] and several novel carotenoids [22] have been identified in *Corbicula*, with carotenoid profiles differing between freshwater and brackish-water clams, presumably reflecting variations in the ingested phytoplankton [22]. These findings indicate that shellfish synthesise endogenous bioactive compounds and bioconcentrate metabolites derived from microorganisms and protists at lower trophic levels. However, detailed studies on *Corbicula* physiology remain limited.

This review focuses on *Corbicula*, especially the Taiwanese freshwater variant, and compiles the available evidence on the biological activities of freshwater clam extract, *shijimi*. Particular emphasis is placed on hepatoprotection, modulation of alcohol metabolism and alcoholic fatty liver, cholesterol-lowering effects, and the mechanisms underlying these effects. This review aims to bridge the gap between traditional ethnomedicinal knowledge and contemporary biomedical research by critically evaluating the pharmacological activities and therapeutic potential of *shijimi*, with a particular focus on hepatoprotection, modulation of alcohol metabolism, lipid regulation, and related mechanisms for the first time. This review is a critical re-evaluation of the existing literature on *shijimi* and is a baseline integrative synthesis of mechanistic insights into the physiological effects of *shijimi* on metabolic syndrome and liver disorders.

The cited studies were reviewed using the keywords ‘freshwater clam’ and ‘metabolism’ in Google Scholar, PubMed, and CiNii Research. The inclusion criteria were determined as follows: Access to the full text, and grey literature was not included.

## 2. Protective Effects in Liver Injury

FCE exhibits prophylactic effects against hepatic injury induced by carbon tetrachloride or haemorrhagic shock [23,24]. The hepatoprotective effects of FCE were further evaluated using a GalN-induced liver injury model [25]. In rats administered FCE, the activities of serum aspartate aminotransferase (AST), alanine aminotransferase (ALT), lactate dehydrogenase, and alkaline phosphatase (ALP) were significantly lower than those in the control group [25]. These findings indicate that *shijimi* effectively suppresses GalN-induced elevation of serum transaminases, reflecting its protective effect against hepatotoxicity.

**Figure 1:**
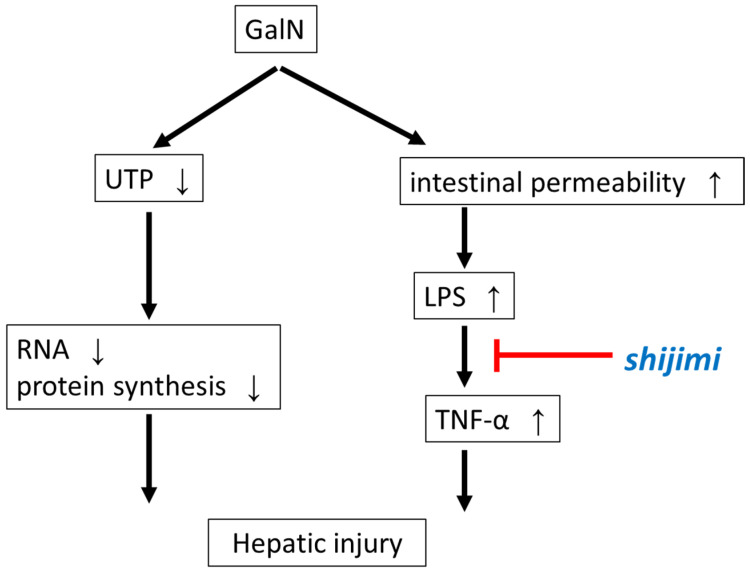
Overview of the protective effect of *Shijimi* against D-galactosamine-induced hepatic injury. GalN: D-galactosamine; UTP: uridine triphosphate; LSP: lipopolysaccharide; TNF-α: tumour necrosis factor-α. Thin arrows; level up (↑) or down (↓).

Nozomu et al. [26] previously fractionated clam extracts into water- and hexane-soluble components and found that the aqueous fraction, either alone or in combination with the lipid fraction, attenuated GalN-induced injury. Comparable findings have been observed in other hepatotoxicity models, such as FCE ameliorating carbon tetrachloride-induced hepatic damage [24] and mitigating haemorrhagic shock-induced injury, with concurrent suppression of tumour necrosis factor-α (TNF-α) production [23,27]. GalN hepatotoxicity is attributed to two non-exclusive mechanisms: (i) GalN is converted to uridine diphosphate-galactosamine, depleting hepatic uridine triphosphate and thereby inhibiting RNA and protein synthesis; and (ii) GalN increases intestinal permeability, facilitating lipopolysaccharide (LPS) translocation and subsequent TNF-α release, leading to hepatocyte apoptosis [10,28]. Although the detailed mechanisms of *shijimi*’s hepatoprotective effects are not fully elucidated, its ability to suppress TNF-α in haemorrhagic shock models suggests a similar anti-TNF-α effect in the GalN model [27] (Figure 1).

Wang et al. [29] reported that glutamine, asparagine, serine, histidine, tyrosine, lysine, and glycine each exert partial protective effects in GalN-induced liver injury. In dietary experiments involving FCE, the extract constituted only 2% of the feed, resulting in no changes in the total amino acid composition compared to the control diets [23]. These observations indicate that free amino acids are unlikely to be responsible for the observed hepatoprotection. The molecular forms, whether proteins or peptides, responsible for the activity of these amino acids in FCE remain undetermined. Clarifying whether specific proteins or peptides in *shijimi* contribute to hepatoprotection remains a challenge.

The FCE contained a relatively high lipid content (approximately 7.2%). Lin et al. [30] separated FCE into a chloroform-soluble lipid fraction (yield: 8.2%) and hot-water-soluble fraction and demonstrated that the lipid fraction inhibited LPS-induced TNF-α production in vitro (in human leukaemia cells and peripheral blood mononuclear cells) and in vivo (in rats). Further detailed investigation is required to determine lipid involvement against GalN-induced injury and TNF-α suppression.

Then, Kuo et al. reported that trans-2-nonadecyl-4-hydroxymethyl-3-dioxolane extracted from freshwater clams alleviates dimethylnitrosamine-induced hepatic fibrosis [31].

In summary, *shijimi* exhibited a protective effect against D-galactosamine-induced hepatic injury. However, the bioactive constituents and detailed molecular mechanisms remain to be elucidated. Future studies using GalN and other hepatotoxic models should focus on isolating active compounds and characterising their pharmacological properties.

## 3. Ethanol-Metabolising and Anti-Steatotic Effects

*Shijimi* have been historically consumed as food and traditional medicine. In the Compendium of Materia Medica (Li Shizhen), clams are described as “dispersing acute heat, brightening the eyes, promoting urination, eliminating heat-related conditions, beriberi and damp toxins, and resolving jaundice caused by alcohol poisoning; its soaked juice cures excessive thirst”. Accordingly, clams have traditionally been regarded as effective in alleviating alcohol toxicity, and clam broth has been empirically used for veisalgia relief. Despite this, scientific investigations on the effects against alcohol-induced hepatic dysfunction remain limited. A representative finding is outlined below.

Aqueous extract of *shijimi* increases alcohol metabolism [32]. The effects of *shijimi* on ethanol-induced hepatic lipid accumulation and blood ethanol concentrations were examined. In rats subjected to acute ethanol dosing, serum AST, ALT, and lactate dehydrogenase (LDH) activities, as well as biomarkers of hepatic injury, showed no significant differences between the FCE-treated and control groups. Similarly, ALP activity, a cholestasis marker, remained unchanged. Serum lipid parameters, including total cholesterol, triacylglycerol, phospholipids, and non-esterified fatty acids, did not show significant differences. Hepatic total lipid and triacylglycerol levels were lower in the *shijimi* group, reaching 76.7% (*p* = 0.127) and 62.0% (*p* = 0.101) of control values, respectively. Hepatic cholesterol content significantly decreased following *shijimi* treatment, reaching 80.1% (*p* < 0.01) of control values.

Blood ethanol concentrations were significantly higher in the FCE group at 2 h after dosing; however, from 5 h onwards, the concentrations were significantly lower at each subsequent time point until ethanol was no longer detectable. In the FCE group, ethanol was cleared from the bloodstream within 10 h, whereas it remained detectable at 11 h in the control group. The area under the concentration-time curve (AUC) was significantly lower in the FCE group [23].

These results indicate that *shijimi* attenuates ethanol-induced hepatic lipid accumulation, significantly reduces hepatic cholesterol levels, and may suppress hepatic fat deposition associated with alcohol consumption. The reduced AUC and more rapid ethanol clearance indicated enhanced ethanol metabolism in FCE-treated rats. Ethanol consumption promotes hepatic fatty acid accumulation by activating the lipogenic transcription factor sterol regulatory element-binding protein 1 (SREBP-1) [33,34]. Although the current study did not examine this pathway under ethanol-loading conditions, previous research has shown that FCE inhibits the expression of lipogenic genes, such as fatty acid synthase (FAS) and stearoyl-CoA desaturase, through SREBP-1 in normal rats [35]. This suggests that a similar mechanism may be activated upon ethanol exposure.

FCE contains abundant free amino acids, including taurine and ornithine, which are associated with improved lipid metabolism [36,37,38,39,40,41]. Nonetheless, the experimental diets in this study contained only trace amounts of taurine (1.38–20.01 mg/100 g) and ornithine (1.02–14.79 mg/100 g) [23], indicating a minimal contribution of these amino acids to the observed effects.

Takeuchi et al. fractionated *Seta* clams into water- and hexane-soluble extracts [42]. Although each fraction alone demonstrated weak activity, their combination showed potential anti-steatotic efficacy in ethanol-induced fatty liver [24]. The active compounds were subsequently identified as α,α′- and α,β-diglycerides of 2-octadecenoic acid [42]. One of the proposed mechanisms of alcohol-induced hepatic injury is the induction of TNF-α production via LPS [43]. Further detailed investigations are warranted to identify the specific bioactive compounds responsible for the ethanol-metabolising and anti-steatotic effects of *shijimi* and elucidate their mechanistic roles in the context of alcohol-induced hepatic stress.

## 4. Cholesterol-Lowering Effect on Diet-Infused Hypercholesterolaemia

Several shellfish species have been reported to exhibit cholesterol-lowering activity [14,42,43,44,45,46,47,48]. In the Japanese basket clam *Corbicula japonica*, consumption was shown to enhance lipid metabolism in rats fed a high-cholesterol diet [15,20]. Iritani et al. demonstrated that the edible portion and its neutral lipid extracts reduced serum and hepatic cholesterol and triacylglycerol levels [15,20]. They proposed that sterols in clams, specifically 24-methylencholesterol and β-sitosterol, may inhibit cholesterol absorption [20].

The cholesterol-lowering effect of FCE was evaluated in rats rendered hypercholesterolaemia by a high-cholesterol, high very low-density lipoprotein diet. FCE administration significantly decreased plasma and hepatic cholesterol levels in hypercholesterolaemic rats [20,49]. These clams contain various sterols capable of competing with cholesterol for intestinal absorption, suggesting a competitive inhibition mechanism. FCE treatment led to dose-dependent increases in faecal plant sterol and neutral sterol excretion [49]. However, it remains unclear whether the phytosterol content alone is sufficient to account for the observed hypocholesterolaemic effects.

Sugano et al. [50] examined the dose dependency of phytosterols in hypercholesterolaemic rats. Based on their findings, the 30% FCE diet used in this study [49] provided sufficient sterols to reduce hepatic cholesterol levels; however, it did not affect serum cholesterol. In addition, the 3% and 15% diets did not contain sufficient sterols to reduce either hepatic or serum cholesterol. Although phytosterols likely contribute, their role appears limited. Nevertheless, FCE markedly increased faecal neutral sterol excretion [49], implying the presence of additional bioactive constituents that facilitate sterol elimination.

Faecal bile acid excretion was elevated in the FCE-treated group [47]. Given that phytosterols do not typically promote bile acid excretion [50,51,52], FCE appears to influence bile acid metabolism, further contributing to its cholesterol-lowering effects. Tanaka et al. [45] reported that oysters increase faecal excretion of neutral sterols and bile acids, possibly via inhibition of bile acid micelle formation in the intestine. This section focuses on FCE, which was shown to induce hepatic cholesterol 7 alpha-hydroxylase (CYP7A1) expression, the rate-limiting enzyme in bile acid biosynthesis, in a dietary hypercholesterolaemia model [49]. This induction may underlie the reduction in serum cholesterol levels.

CYP7A1 expression is regulated by bile acids via small heterodimer partner (SHP)-mediated repression, whereas hepatocyte nuclear factor-4 (HNF-4) and liver X receptor (LXR) mediate its positive regulation [53]. FCE-induced CYP7A1 upregulation occurs without significant changes in SHP mRNA expression [49]. Further analysis revealed increased HNF-4 mRNA levels, whereas LXR remained unchanged. LXR activates CYP7A1 by binding to the direct repeat (DR-4) element [54], whereas HNF-4 binds to the DR-1 element in the bile acid-responsive element-II, enhancing promoter activity [55,56]. Hence, CYP7A1 induction by FCE may be mediated through HNF-4 upregulation. Although HNF-4 regulates apolipoprotein (apoA-I), the major protein component of high-density lipoprotein [57], FCE increased HNF-4 expression without affecting apoA-I mRNA levels [49], suggesting that additional regulatory mechanisms may contribute to CYP7A1 expression.

**Figure 2:**
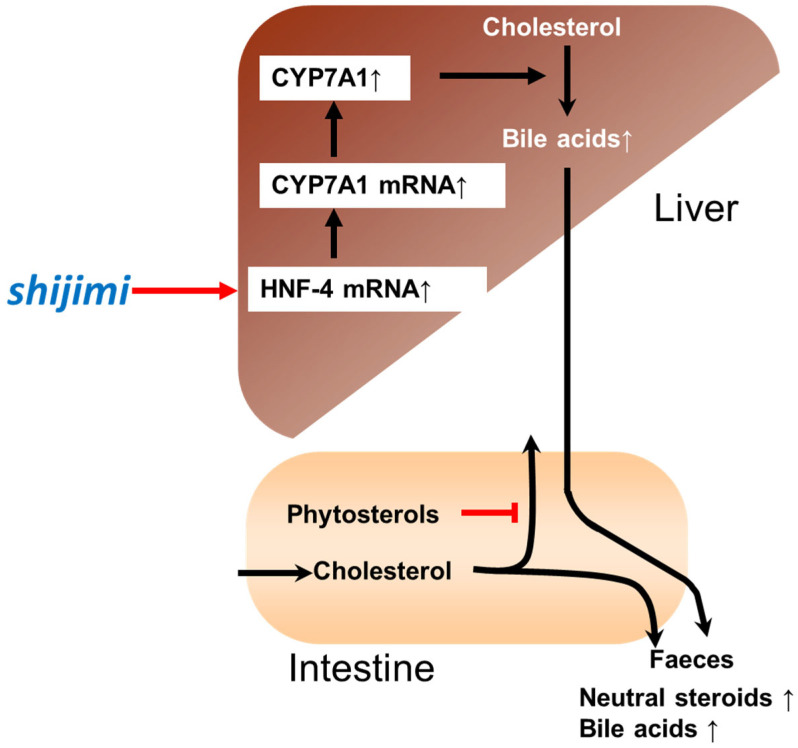
Mechanism of the cholesterol-lowering effect of *Shijimi.* CYP7A1: cholesterol 7 alpha-hydroxylase; HNF-4: hepatocyte nuclear factor-4. Thin arrows; level up (↑) or down (↓).

In summary, *shijimi* may have cholesterol-lowering effects through multiple mechanisms. In addition to competitive inhibition of cholesterol absorption by phytosterols, *shijimi* may appear to enhance sterol excretion and bile acid synthesis, potentially mediated via CYP7A1 induction through HNF-4 activation (Figure 2).

## 5. Cholesterol-Lowering Active Compounds on Diet-Infused Hypercholesterolaemia

### 5.1. Cholesterol-Lowering Effect of the Lipid and Isolated-Protein Fractions of Freshwater-Clam

This section focuses on the major components of FCE, lipids and proteins, and summarises the findings of studies involving hypercholesterolaemic rats administered these fractions. Both lipid and isolated protein fractions significantly reduced serum and hepatic cholesterol concentrations [58], with the lipid fraction demonstrating a more pronounced effect.

Ikeda et al. [59] reported that soy protein upregulates hepatic ATP-binding cassette subfamily G member 5 (ABCG5) and ABCG8, thereby enhancing biliary cholesterol secretion without affecting faecal neutral sterol excretion. Similarly, the FCE protein fraction induced ABCG5 expression without altering faecal neutral sterol output [58]. Higaki et al. [60] proposed that bile acids may bind to peptides in soy proteins and be excreted in the faeces. In the whole FCE and protein fraction groups, faecal volume significantly increased compared to that in the controls [58], indicating the presence of poorly digestible proteins. However, bile acids bound to hydrophobic substances in faeces were not elevated in the protein fraction group [58]. FCE upregulated hepatic CYP7A1 expression without influencing SHP, a negative regulator of CYP7A1 via the farnesoid X receptor [58,61,62]. Therefore, hydrophobic compounds in the protein fraction are unlikely to mediate bile acid excretion or contribute directly to cholesterol reduction.

The amino acid composition of FCE plays an important role in cholesterol metabolism [7]. FCE contains approximately two- to threefold higher levels of glycine than casein, whereas levels of sulphur-containing amino acids are comparable [58]. Glycine and sulphur amino acids have been reported to reduce plasma cholesterol in hypercholesterolaemic rats [62,63]. Although the exact mechanism underlying the hypocholesterolaemic effect of FCE remains unclear, its specific amino acid profile and unidentified bioactive peptides may contribute to this activity.

Both lipid and protein fractions of FCE exhibited cholesterol-lowering activity. Additionally, FCE may confer benefits in metabolic syndrome, hepatic injury, and cholestasis. The lipid fraction suppressed serum ALT activity, a marker of hepatic injury. Serum levels of direct bilirubin and ALP, as well as indicators of cholestasis induced by dietary cholesterol, were attenuated by FCE supplementation. Metabolic syndrome, typically associated with obesity, is characterised by insulin resistance and chronically elevated plasma free fatty acid levels [64]. In rats administered whole *shijimi*, its lipid fraction, or its protein fraction, serum free fatty acid concentrations decreased, and serum adiponectin levels increased [58]. These findings suggest that FCE has therapeutic potential for the management of metabolic syndromes and other lifestyle-related diseases.

### 5.2. Cholesterol-Lowering Effect of the Lipid Fractions of Freshwater-Clam

Given the rising prevalence of hypercholesterolaemia and interest in functional foods, this section outlines the cholesterol-lowering constituents of the lipid fraction of FCE. Thin-layer chromatography identified a broad spectrum of lipid components. Maoka et al. [22] reported the isolation of 43 novel carotenoids from clams.

**Figure 3:**
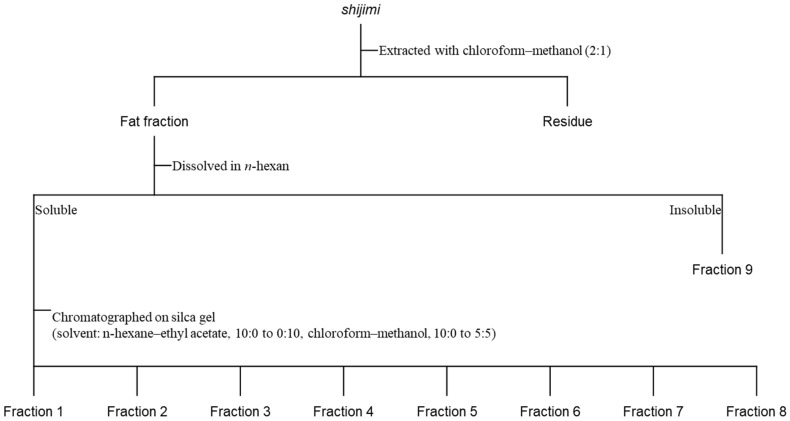
Preparation scheme of the fat subfraction of Shijimi. Fraction 1 shows the most pronounced activity and upregulated hepatic CYP7A1 expression. Fraction 2 exhibits notable cholesterol-lowering activity. Most phytosterols are concentrated in Fraction 4.

In a study published by Chijimatsu et al. [65], the lipid fraction was fractionated into nine subfractions using silica gel column chromatography to isolate bioactive components (Figure 3). In hypercholesterolaemic rats, significant cholesterol-lowering effects were observed in subfractions rich in sphingolipids, triacylglycerols, and sterol esters. Among these, Fraction 1, which contained a high concentration of sphingolipids, exhibited the most pronounced activity and significantly upregulated hepatic CYP7A1 expression, suggesting that sphingolipids enhance the hepatic conversion of cholesterol to bile acids. Fraction 2, composed mainly of triacylglycerols and sterol esters, exhibited a notable cholesterol-lowering activity. Although phytosterols were present in this fraction, their concentration was approximately one-thirtieth of that in Fraction 4, which lacked cholesterol-lowering activity, indicating that phytosterols likely play a minor role. Previous studies by Iritani et al. [20] and Sugano et al. [50] attributed the hypocholesterolaemic effect of clams to phytosterols; however, Chijimatsu et al. [65] reported that the levels present in *shijimi* were insufficient to account for their efficacy. Most phytosterols were concentrated in Fraction 4, which did not exhibit significant activity. Although Fraction 4 increased faecal neutral sterol excretion [65], this suggests that phytosterols may primarily contribute to sterol elimination rather than being the principal hypocholesterolaemic mechanism of the lipid fraction. Furthermore, the lipid fraction reduced biomarkers associated with cholestasis and metabolic syndrome. It attenuated diet-induced elevation of cholestasis markers and suppressed serum free fatty acid levels. Considering the established link between metabolic syndrome, insulin resistance, and elevated free fatty acid levels [62], these findings have possibilities of the potential therapeutic relevance of FCE in managing metabolic disorders.

In conclusion, active fractions of sphingolipids, triacylglycerols, and sterol esters lower cholesterol through mechanisms distinct from those traditionally associated with these lipid classes.

## 6. Cholesterol-Lowering Effect on Xenobiotic-Infused Hypercholesterolaemia

Dietary (exogenous) cholesterol constitutes approximately 20% of total body cholesterol in humans, with the remaining 80% synthesised endogenously in the liver. Consequently, hepatic cholesterol synthesis disorders warrant clinical attention. Xenobiotics promote hypercholesterolaemia by stimulating hepatic cholesterol biosynthesis. In a chloretone-induced endogenous hypercholesterolaemia rat model, *shijimi* demonstrated potent cholesterol-lowering effects [66]. This effect was associated with upregulation of hepatic CYP7A1 and increased faecal excretion of neutral sterols and bile acids, indicating enhanced cholesterol catabolism to bile acids. Expression of 3-hydroxy-3-methylglutaryl-coenzyme A reductase remained unaltered. Therefore, *shijimi* reduced cholesterol predominantly by promoting catabolic pathways in both exogenous and endogenous hypercholesterolaemic models.

*Shijimi* mitigated chloretone-induced hepatic lipid accumulation and cytotoxicity. Both the lipid and isolated protein fractions exhibited similar hypolipidaemic and hepatoprotective effects, with the lipid fraction showing particular potency [66]. Xenobiotics induce fatty liver mainly by selectively activating hepatic NADPH-generating enzymes, including glucose-6-phosphate dehydrogenase (G6PD), malic enzyme (ME), and 6-phosphogluconate dehydrogenase [67]. Transcriptomic analysis implicated the modulation of genes regulating fatty acid synthesis and signalling in the triglyceride-lowering effect of FCE [35], identifying SREBP-1 as a key transcriptional regulator and confirming alterations in several lipid metabolic genes [35].

Chloretone, polychlorinated biphenyls (PCBs), butylated hydroxytoluene, and other xenobiotics induce cytochrome P450 enzymes [68] that generate reactive oxygen species (ROS), such as superoxide and hydrogen peroxide [69]. Xenobiotic exposure triggers hepatic lipid peroxidation [70], contributing to hepatotoxicity. Although chloretone induced hepatic cytochrome P450 isoforms 2B1 and 2B2 (CYP2B1/2B2) expression, *shijimi* did not modulate these isoforms [66], indicating that hepatoprotection is independent of CYP2B modulation. Oral FCE administration suppressed hepatic lipid peroxidation in CCl4-treated rats [24], whereas vitamin E, despite reducing peroxidation, failed to ameliorate PCB-induced hepatic injury [71,72]. These findings indicate lipid peroxidation may not be the primary mechanism of xenobiotic hepatotoxicity. ROS activate nuclear factor kappa-light-chain-enhancer of activated B cells, which upregulates pro-inflammatory gene expression [73,74]. FCE suppressed TNF-α production and hepatic injury in a haemorrhagic shock model [24]. Unpublished transcriptomic data further demonstrated that *shijimi* and its lipid and protein fractions attenuated chloretone-induced hepatic TNF-α mRNA elevation, implicating TNF-α suppression in the hepatoprotective mechanism of *shijimi*.

## 7. Cholesterol-Lowering Active Compounds on Xenobiotic-Induced Hypercholesterolaemia

Iritani et al. [15] reported that neutral lipids from *C. japonica* suppressed hepatic G6PD and ME activities; these lipids comprised >35% long-chain polyunsaturated fatty acids (LC-PUFAs; C18:4, C20:5, and C22:6). LC-PUFAs of the n-3 series inhibit TNF-α and confer protection in non-genetic models of non-alcoholic steatohepatitis [75]. However, in the *shijimi* lipid fraction, oleic and linoleic acid levels were only 1⁄13 and 1⁄50 of those in corn oil, respectively, and LC-PUFAs were not abundant [66]. Despite this, the fraction reduced lipid accumulation and hepatic injury, suggesting that lipophilic bioactive constituents other than LC-PUFAs mediate these effects.

Dietary protein and amino acid composition influence hepatic fatty acid synthesis [76,77,78], and certain amino acids possess hepatoprotective activity [79]. Although the amino acid profile of FCE closely resembles that of casein, its glycine content is two- to threefold higher [64]. Glycine regulates lipogenesis [8,9] and attenuates D-galactosamine-induced hepatic injury, partly by suppressing TNF-α release from Kupffer cells [10,80]. Whether the elevated glycine content contributes to the lipid-lowering and hepatoprotective effects of FCE proteins remains to be determined.

Plant-derived proteins (such as wheat gluten and soy) suppress hepatic triacylglycerol accumulation relative to animal proteins and are associated with elevated G6PD and ME activities [76]. Soy protein enhances lipogenic gene expression [77]; however, it ameliorates steatosis via SREBP-1 suppression [81]. An amino acid mixture simulating soy composition suppresses G6PD and ME without altering hepatic triglyceride levels [76]. These findings suggest that the cholesterol-lowering effect of FCE protein is likely mediated through mechanisms independent of its amino acid composition.

A salient difference between the chloretone- and galactosamine-induced hepatic injury models is the presence of steatosis. Owing to changes in lifestyle, metabolic syndrome and its hepatic phenotypes, including non-alcoholic fatty liver disease and non-alcoholic steatohepatitis (NASH), have emerged as major health concerns. The two-hit hypothesis of NASH posits hepatic fat accumulation as the first hit, followed by oxidative stress and cytokine-mediated inflammation [82]. FCE prevents hepatic injury and inhibits lipid deposition. Both lipid and protein fractions attenuated hepatic lipid accumulation, indicating the presence of bioactive water- and lipid-soluble compounds capable of modulating multiple stages of NASH pathogenesis, including steatosis and cytokine production.

FCE and its lipid and protein fractions reduced serum cholesterol levels in rats with chloretone-induced hypercholesterolaemia [64]. Three complementary mechanisms have been proposed: (i) enhanced hepatic catabolism of cholesterol to bile acids, (ii) increased biliary cholesterol excretion, and (iii) promotion of faecal excretion of neutral sterols and bile acids. The expression of CYP7A1, the rate-limiting enzyme in bile acid synthesis, was modestly induced. The lipid fraction augmented faecal excretion of both neutral sterols and bile acids, whereas the protein fraction selectively increased bile excretion [64]. Similar patterns have been observed in dietary models of hypercholesterolaemia.

Finally, *shijimi* suppressed the hepatic expression of FAS and FADS1, whereas the protein fraction showed no such effect [66], indicating differential gene targets for each fraction. Collectively, *shijimi* contains multiple active constituents that modulate cholesterol metabolism, attenuate steatosis, and confer hepatoprotection through distinct and complementary mechanisms.

## 8. Conclusions and Future Perspectives

Metabolic syndrome is a significant risk factor for atherosclerotic diseases, including cardiovascular and cerebrovascular disorders. Its hepatic manifestations include MASLD and MASH, whereas alcoholic steatohepatitis (ASH) may progress to fibrosis, cirrhosis, and hepatocellular carcinoma. Factors such as diet, physical inactivity, alcohol consumption, smoking, and inadequate rest are closely associated with the development and progression of these conditions. Although habit modification, such as improving diet quality and increasing physical activity, remains the preferred strategy, maintaining a healthy lifestyle remains challenging. Disruption of circadian rhythms in night-shift and rotating-shift workers further exacerbates lifestyle-related diseases [83]. Therefore, pharmacological agents and functional foods serve as valuable adjunctive interventions.

Statins, globally used for dyslipidaemia management, were originally isolated from *Penicillium citrinum* [84], and paclitaxel, an anticancer agent, was derived from the yew tree (*Taxus* spp.) [85]. Although terrestrial organisms have long been the primary source of pharmaceuticals, research has increasingly targeted aquatic organisms through marine biotechnology. Their distinct habitats and metabolic pathways yield potential for the development of novel bioactive compounds. Microalgae and aquatic bacteria alone comprise over 100,000 species; however, direct screening is impeded by the low levels of bioactive substances present in individual organisms. Filter-feeding shellfish, such as clams, accumulate trace lipophilic metabolites produced by these microorganisms and thus constitute advantageous targets for bioprospecting.

This review collates studies on the Taiwanese freshwater clam (*Corbicula fluminea*) and its hot-water extract, organisms characterised by extensive dietary exposure yet limited scientific investigation. *Shijimi* may have effects on lifestyle-related diseases and the identification of their active constituents. Evidence may suggest the capacity of *shijimi* to mitigate hepatic disorders arising from lifestyle factors, including ASH and MASLD/MASH, and to reduce hypercholesterolaemia, a key risk factor for atherosclerosis. FCE has been confirmed to be safe for liver function [86]. Moreover, cholesterol-lowering constituents of *shijimi* appear to act via mechanisms distinct from those of statins, suggesting novel bioactivities. These findings underscore the efficacy of *shijimi* in mitigating hepatic dysfunction and lipid dysregulation, implicating multiple active constituents.

Building on the foundational knowledge outlined above, many bioactivities of *shijimi* have been reported. This indicates that the functionality is exerted as a synergistic effect of multiple ingredients, rather than a single ingredient acting as in medicine. Although the mechanisms of action of these molecules are different, detailed mechanistic insights remain incomplete. It is even possible that additive or synergistic effects may occur by acting on multiple points of action as a food. This review consolidates evidence supporting hepatoprotective and cholesterol-lowering effects of *shijimi*; however, the precise molecular pathways and active constituents responsible for these effects warrant further targeted investigation. Moreover, the differential impacts of *shijimi* components in exogenous versus endogenous hypercholesterolaemia models highlight the complexity of their action and suggest areas for deeper exploration. These observations underscore the need for future studies to isolate and characterise specific bioactive molecules and clarify their mechanistic roles in xenobiotic-induced hepatic stress, thereby advancing our currently fragmented understanding. However, the findings presented in this review are merely suggestions of possibilities based on a fragmented understanding. Therefore, future clinical trials involving humans will be necessary to integrate this fragmented understanding and clarify the effects.

The total synthesis of natural products is often challenging; alternatively, the cultivation of the source organism as a “biological factory” can facilitate the supply of target compounds or precursors. Although the cultivation of deep-sea organisms remains challenging [87], the aquaculture of *C. fluminea* is well-established.

If useful compounds are discovered through further research, it is highly possible that they can be produced in a biological factory. The useful compounds obtained from the *C. fluminea* plant will be used to develop functional foods and medicines for lifestyle-related diseases, leading to an extension of healthy life expectancy while maintaining quality of life.

## Data Availability

No new data were created or analysed in this study.

## Abbreviations

The following abbreviations are used in this manuscript:

MASLD	metabolic dysfunction-associated steatotic liver disease
MASH	metabolic dysfunction-associated steatohepatitis
FCE	freshwater clam extract
GalN	D-galactosamine
AST	aspartate aminotransferase
ALT	alanine aminotransferase
ALP	alkaline phosphatase
TNF-α	tumour necrosis factor α
LPS	Lipopolysaccharide
AUC	area under the concentration-time curve
SREBP 1	sterol regulatory element-binding protein 1
FAS	fatty acid synthase
CYP7A1	cholesterol 7 alpha-hydroxylase
SHP	small heterodimer partner
HNF-4	hepatocyte nuclear factor-4
LXR	liver X receptor
DR	direct repeat
apo	Apolipoprotein
ABCG5	ATP-binding cassette subfamily G member 5
G6PD	glucose-6-phosphate dehydrogenase
ME	malic enzyme
PCBs	polychlorinated biphenyls
ROS	reactive oxygen species
LC-PUFAs	long-chain polyunsaturated fatty acids
NASH	non-alcoholic steatohepatitis
ASH	alcoholic steatohepatitis
